# Disrupted Structural and Functional Networks and Their Correlation with Alertness in Right Temporal Lobe Epilepsy: A Graph Theory Study

**DOI:** 10.3389/fneur.2017.00179

**Published:** 2017-05-03

**Authors:** Wenyu Jiang, Jianping Li, Xuemei Chen, Wei Ye, Jinou Zheng

**Affiliations:** ^1^Department of Neurology, The First Affiliated Hospital of Guangxi Medical University, Nanning, China; ^2^Department of Radiology, The First Affiliated Hospital of Guangxi Medical University, Nanning, China

**Keywords:** temporal lobe epilepsy, alertness, structural network, functional network, graph theory analysis

## Abstract

Previous studies have shown that temporal lobe epilepsy (TLE) involves abnormal structural or functional connectivity in specific brain areas. However, limited comprehensive studies have been conducted on TLE associated changes in the topological organization of structural and functional networks. Additionally, epilepsy is associated with impairment in alertness, a fundamental component of attention. In this study, structural networks were constructed using diffusion tensor imaging tractography, and functional networks were obtained from resting-state functional MRI temporal series correlations in 20 right temporal lobe epilepsy (rTLE) patients and 19 healthy controls. Global network properties were computed by graph theoretical analysis, and correlations were assessed between global network properties and alertness. The results from these analyses showed that rTLE patients exhibit abnormal small-world attributes in structural and functional networks. Structural networks shifted toward more regular attributes, but functional networks trended toward more random attributes. After controlling for the influence of the disease duration, negative correlations were found between alertness, small-worldness, and the cluster coefficient. However, alertness did not correlate with either the characteristic path length or global efficiency in rTLE patients. Our findings show that disruptions of the topological construction of brain structural and functional networks as well as small-world property bias are associated with deficits in alertness in rTLE patients. These data suggest that reorganization of brain networks develops as a mechanism to compensate for altered structural and functional brain function during disease progression.

## Introduction

Recently, complex networks theory has been widely used to investigate the structure and function of the human brain. The dynamics and architecture of brain complex networks change in a particular pathophysiological status. Further, changes in brain complex networks probably cause brain dysfunction. A complex network that is described mathematically by graph theory, such as a brain network based on neuroimaging or electroneuro-physiology data, has the capability to provide valuable insights into not only the architecture of the whole-brain networks (1) but also their potential relation with decline in cognitive comorbidity (2).

Temporal lobe epilepsy (TLE) is the most common type of focal epilepsy in adults. From the viewpoint of the generation and propagation mechanisms of seizures, TLE could be considered a “network disease” (3). TLE is mainly associated with hippocampal sclerosis and other temporal lobe lesions, such as isolated amygdala abnormalities (4). However, evidence from diffusion tensor imaging (DTI) studies has shown that white matter microstructure damage often extends beyond epileptic lesions, such that the distal part of nerve fiber bundles is also affected (5, 6). Moreover, functional connectivity (FC) abnormalities in epileptogenic networks have been observed in fMRI and electrophysiological studies (7, 8). Therefore, focal seizures originate in the abnormal epileptic network rather than from localized lesions (9) and spread through nerve bundles to distal regions of the whole brain, resulting in injuries to extra-temporal lobe areas (2) These injuries were found to be associated with TLE patients’ brain dysfunction (5, 6).

Although some TLE patients have normal intelligence, some patients exhibit domain-specific cognitive impairment, such as naming difficulty, memory loss, and executive dysfunction, among other impairments. These domain-specific cognitive deficits have been attributed to damage to the structure or function of the temporal lobe or to the development of abnormal epileptic networks within or external to the temporal lobe (3, 5). Although lesions outside the temporal lobe may not be detectable under a 3.0-T structural Magnetic Resonance Imaging scan, patients’ cognitive decline or psycho-behavioral abnormalities can usually be detected (10, 11). Lately, these types of brain dysfunctions have often been studied and explained by using graph theoretical analysis of complex networks.

Alertness in brain function is characterized as having a high sensitivity to an incoming stimulus and maintaining this state of a high-sensitivity level to respond in time. It is a prerequisite for more complex and capacity-demanding components of attention, such as selectivity, which is a fundamental component of attention (12). Attention deficit is a common symptom in patients with mesial temporal lobe epilepsy (13). Obviously, considering that attention is one of the core functions of cognition (14), it is important to study alertness. According to Posner, the alertness network may depend on the right frontal and parietal lobes as well as the locus coeruleus (12). In line with Posner’s model, neuroimaging studies have found that the alerting network involves the right frontal and posterior parietal areas and is probably modulated by the norepinephrine system (15, 16). Many clinical studies have investigated patients with right parietal lesions who have difficulty in maintaining a state of attention and use warning signals to improve performance (17). Patients with right hemisphere stroke have a particular difficulty in sustaining a high level of alertness, although alertness recovers after alertness training (18). These results support the involvement of the predominantly right-side fronto-parieto-thalamic network in controlling alertness. Additionally, cognitive control of alertness relies on a predominantly right hemisphere cortical and subcortical network (19). Our previous task-based fMRI study indicated that activation of alertness-related brain areas, such as the right occipital and right frontal lobe, was significantly attenuated in right temporal lobe epilepsy (rTLE) patients (20). Extension of resting-state functional MRI (rsfMRI) findings in rTLE patients demonstrated that decreased FC between the right thalamus, anterior cingulate cortex (ACC) (21), and right cuneus (22) was correlated with the alertness.

Based on the above hypotheses, clinical observations, and neuroimaging studies, we hypothesize that neural circuits in the right hemisphere, including the dorsolateral prefrontal cortex, ACC, inferior parietal cortex, and thalamus, are involved in TLE (17). Therefore, we further hypothesize that some pathophysiological alterations occur in the right hemisphere in brain networks of TLE patients and that these changes are related to the underlying changes of alertness. Studying right TLE patients may be more sensitive in its ability to detect a relationship between alertness and the organization of brain networks. For this purpose, we constructed white matter structural networks by using DTI tractography and established functional networks from rsfMRI temporal series of rTLE patients and healthy controls. Next, we performed comparative calculations to identify group differences of topological parameters. Correlation analyses between alertness and network organization were performed to detect the underlying relational mechanism.

## Materials and Methods

### Subjects

Twenty rTLE patients (10 females, 10 males, age 26.35 ± 5.97) were recruited consecutively from Epilepsy Clinic, the First Affiliated Hospital of Guangxi Medical University according to the diagnostic manual of the International League Against Epilepsy classification (23). They were recruited from July 2015 to May 2016. All patients underwent standard clinical assessments, including a detailed seizure history, neurological examination, neuropsychological assessment, standard and video-EEG evaluation, and brain MRI. Particularly, all rTLE patients met at least two of the following criteria (24): (1) the typical symptoms of TLE indicated that the epileptogenic lesion was located in the temporal lobe; (2) MRI showed right hippocampus atrophy, sclerosis, or other abnormality of the right temporal lobe. All of the images were assessed by a neuroradiologist. (3) Electroencephalogram (EEG) revealed ictal or interictal discharges in the right temporal lobe, as evaluated by an epilepsy specialist. Our patients had taken regular antiepileptic drugs (AEDs) and had no epileptic seizures in the last 3 months. Additionally, to avoid any confounding effects on cognition, we excluded patients with a Mini-Mental State Examination (MMSE) score <24 as well as any history of neurological or psychiatric disorder other than TLE, traumatic brain injury, or other serious disease.

The control group consisted of 19 age-, gender-, and mean educational years-matched healthy volunteers (9 females, 10 males, age 26.47 ± 3.78). All had no history of neurological or psychiatric disorders. This research was approved by the Ethics Committee of the First Affiliated Hospital of Guangxi Medical University. All participants were right handed and provided signed informed consent prior to the study.

### Data Acquisition

All MR images were performed on a 3-T Achieva MRI scanner (Philips, Netherlands) with a 12-channel phased array head coil. A 3D high-resolution structural image was acquired for each subject with a T1-weighted spin-echo sequence (TR/TE = 3,000/10 ms, slice thickness = 5 mm, slice gap = 1 mm) for spatial brain normalization.

Diffusion tensor imaging images were acquired using a single-shot echo-planar imaging-based sequence with the following parameters: TR/TE = 6,100/93 ms, flip angle = 90°; FOV = 240 mm × 240 mm, slice thickness = 2 mm and no gap, number of signals acquired = 4; data matrix = 256 × 256, flip angle = 90°, voxel size = 0.94 mm × 0.94 mm × 3 mm; resulting in a total of 30 volumes with diffusion gradients applied along 30 non-linear directions (*b* = 1,000 s/mm^2^) and 1 volume without diffusion weighting (*b* = 0 s/mm^2^). Each volume consisted of 45 contiguous axial slices.

The rsfMRI data were obtained using a gradient-echo echo-planar imaging sequence with parameters of: TR/TE = 2,000/30 ms, flip angle = 90°, FOV = 220 mm × 220 mm, data matrix = 64 × 64, slice thickness = 5 mm, slice gap = 1 mm, and voxel size = 3.44 mm × 3.44 mm × 6.00 mm; 31 slices and 180 volumes were acquired. All participants were instructed to lie still while resting with their eyes open and were forbidden to think of anything in particular.

### Data Processing

The DTI data were preprocessed using PANDA^1^ (25) in Matlab including the following steps: converting DICOM files into NIfTI images, estimating the brain mask, cropping the raw images, correcting for the eddy current effect, correcting for head motions, estimating the diffusion tensor models by using the linear least-squares fitting method on each voxel, tracking whole-brain fiber in the native diffusion space *via* Fiber Assignment by using the Continuous Tracking algorithm, and averaging multiple acquisitions and calculating diffusion tensor metrics.

The fMRI data were preprocessed using SPM8^2^ and the GRETNA toolbox^3^ (26). The preprocessing steps included removal of volumes, slice timing correction, realignment, spatial normalization, and temporal filtering as follows. The first 10 volumes of each subject were removed to ensure magnetization equilibrium. The remaining volumes were then executed for slice timing correction based on the middle slice and then realigned for head motion correction. Two patients were excluded from further calculations due to head motion >2 mm or head rotations <2°. For group average and group comparison purposes, the data were spatially normalized to the standard Montreal Neurological Institute space and resampled with a resolution of 3 mm × 3 mm × 3 mm. Subsequently, signals were typically band-pass (0.01–0.08 Hz) filtered to reduce the effects of low-frequency drift and high-frequency physiological noise (27). Finally, confounding variables, including six head motion parameters, averaged global and white matter signals, and cerebrospinal fluid regressed out.

### Construction of Brain Networks

The nodes of the structural and FC networks were delimited according to an automated anatomical labeling (AAL) algorithm (AAL) algorithm (28). This algorithm scheme parcellated the entire cerebral cortex, except the cerebellum, into 90 anatomical regions (AAL-90), which resulted in 90 nodes covering the non-cerebellar brain and 45 nodes in each hemisphere.

#### Structural Network Construction

Structural networks were constructed using deterministic tractography using the PANDA toolbox. A FA-weighted matrix (90 × 90) generated from PANDA was thresholded into different levels to create an adjacency matrix. Each matrix represented the white matter network of the cerebral cortex, in which each row or column represented a brain region of the automated anatomical labeling template. For each subject, the FA-weighted matrix was used for further graph analyses.

#### Functional Network Construction

Functional networks were constructed orderly using the GRETNA toolbox. A 90 × 90 temporal correlation matrix was assembled by computing Pearson’s correlation coefficient between the residual time series of each pair of the 90 nodes for each participant. For each ROI, the mean time series was obtained by averaging the fMRI time courses over all regions. The values of the inter-regional correlation coefficients were taken as the weights of the edges. Thus, we constructed a weighted symmetric FC matrix for each participant. Because of the multiple, non-independent comparisons entailed by thresholding each of inter-regional correlations, we built the FC matrix using a FDR 0.05-corrected threshold (29). Based on this weighted FC matrix, the topological properties of the network were subsequently calculated by graph theoretic analyses.

### Graph Analysis

Graph theoretical analyses of the weighted structural and functional networks of rTLE patients and controls were calculated with routines from the GRETNA toolbox. The network topological properties at the global levels were collected, including (1) properties that imply network segregation of brain, such as the weighted clustering coefficient (γ), local efficiency (E_loc_), and modularity; (2) properties that indicate network integration of the brain, such as the characteristic path length (λ) and global efficiency (E_glob_). E_glob_ is defined as the average inverse shortest path length; E_loc_ is defined as the mean of the global efficiencies of subgraphs consisting of the immediate neighbors of a particular node (30). (3) Small-worldness (σ) which evaluates the balance of segregation and integration.

Network topological properties rely on the density of network. So the difference of connectivity strength may affect networks comparisons. When brain graphs constructed, each graph of subjects was thresholded to create an equal number of nodes and edges across subjects (31). We operated network parameters over a range of threshold values to guarantee high correlation coefficients of the remaining connections. As in DTI, we used FA as the value for threshold. And in fMRI, we used the concept of sparsity to analyze the network. The sparsity was defined as a density range of 0.2–0.42, since these densities provide a reasonable trade-off between sparse, but not fully connected networks and highly linked networks, which do not show small-world properties any more (32, 33).

### Neuropsychological Test of Alertness

Each participants’ alertness was assessed by attention network test (ANT) (34) based on the E-Prime software platform. The ANT is a common neuropsychological examination that is used to test for attentional deficits and is composed of the Flanker task and cued response time (RTs) task. Alertness is one of the three basic components of attentional function. Through this examination, alertness can be detected by changing the cue prompt and recording both correct and incorrect reactions as well as the reaction time. The correct reaction and its RTs were applied to evaluate alertness according to the formula: mean RT_no cue_ − mean RT_double cue_. Based on the classification of alertness (12), the no cue condition expresses intrinsic alertness and double cue condition represents phasic alertness. To reduce the effect of executive control function on the intrinsic and phasic alertness RTs, we excluded in-congruent trials during the calculation (35).

### Statistical Analysis

Statistical analyses were performed by using IBM SPSS statistics (version 22). A two-sample *t*-test was performed to analyze group differences in age, years of education, MMSE scores, and ANT scores of alertness between rTLE patients and controls. The Chi square test was employed to compare gender distributions between groups, and *p*-value less than 0.05 was considered statistically significant.

The graph measurements, such as γ, λ, σ, E_loc_, and E_glob_ were analyzed with ANCOVA to detect differences between rTLE patients and controls over a wide range of threshold, with FDR correction. Age and gender were included in ANCOVA as nuisance covariates.

Pearson correlation analyses were performed to assess the correlations between alertness and graph theoretical measures of the structural and functional networks at each FA or the sparsity threshold value. Considering that the disease duration of epilepsy might be a confounding factor, we performed partial correlation analysis to remove the interference. As these analyses were exploratory in nature, we used a statistical significance level of *p* < 0.05, uncorrected.

### Network Visualization

The resultant group level structural and functional networks were displayed using Pajek.^4^

## Results

### Demographic Characteristics

There were no significant difference in age, sex, and educational level between the rTLE group and normal control group. The demographic details of the participants are summarized in Table 1. rTLE patients were treated with drugs without surgery; both groups of subjects were right handed.

**Table 1:** **Demographic characteristics of rTLE participants and controls**.

Characteristics	rTLE (*n* = 18)	Control (*n* = 19)	*t*/χ^2^ value	*p*-Value
Age (years)	26.35 ± 6.12	26.47 ± 3.78	0.075	0.940^a^
Gender (male/female)	9/9	9/10	0.027	0.869^b^
Education (years)	12.3 ± 1.92	12.79 ± 1.36	0.367	0.914^a^
Handedness (right/left)	18/0	19/0	n.a	n.a
Duration of epilepsy	8.68 ± 5.99	–	n.a	n.a
Mini-Mental State Examination	27.6 ± 0.99	27.89 ± 0.74	1.05	0.302^a^

*Age of participants and duration of the epilepsy are shown with mean ± SD*.

*rTLE, right temporal lobe epilepsy; n.a, not applicable*.

*^a^Obtained by a two-sample two-tailed t-test*.

*^b^Obtained by a two-tailed Pearson’s χ^2^-test*.

### Global Topology of Structural and Functional Networks

**Figure 1:**
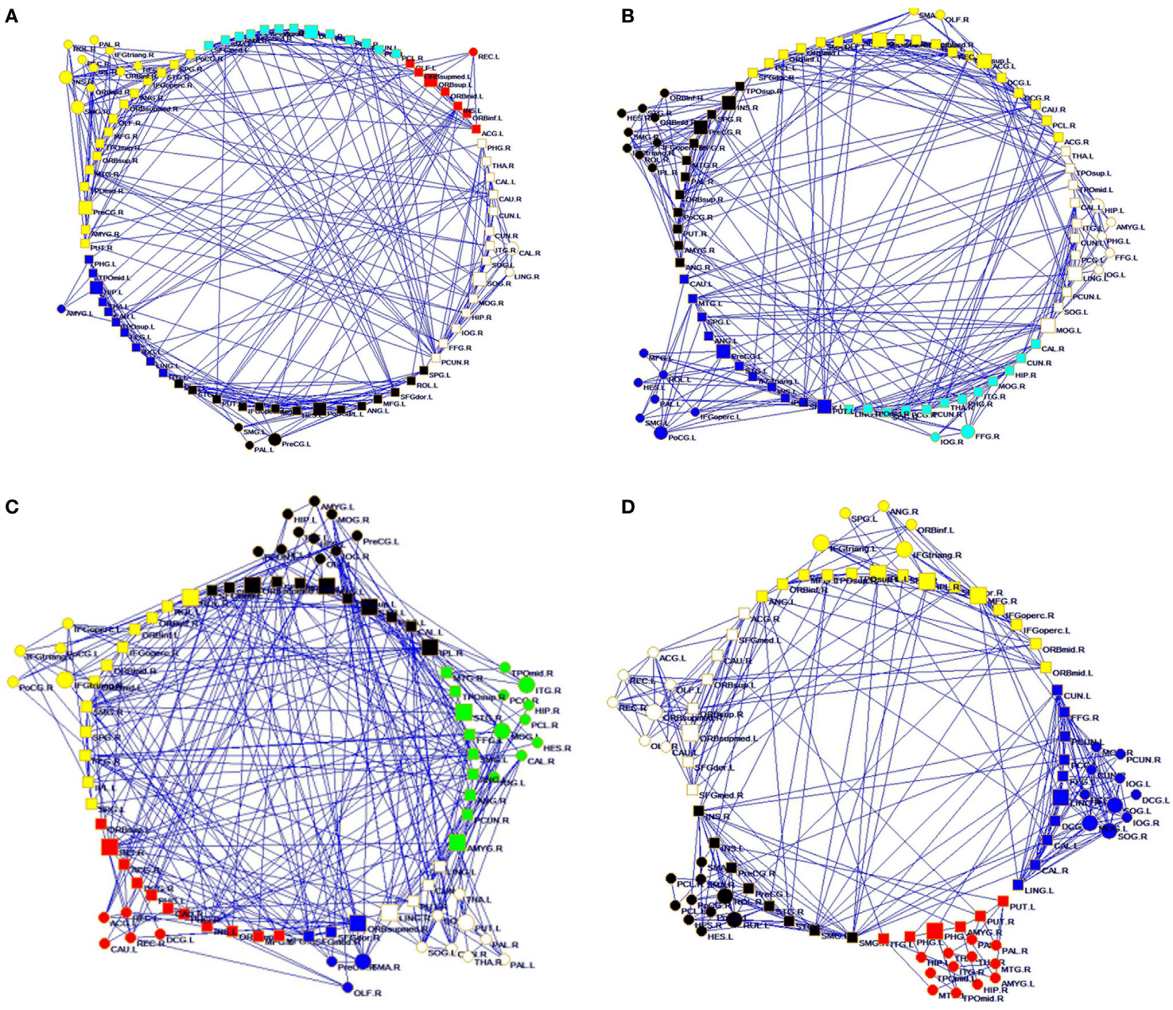
**Structural connectivity network and functional connectivity network visualization of controls (A,C) and right temporal lobe epilepsy patients (B,D)**. **(A,B)** Structural network at FA = 0.28; **(C,D)** functional network at sparsity = 0.08.

**Figure 2:**
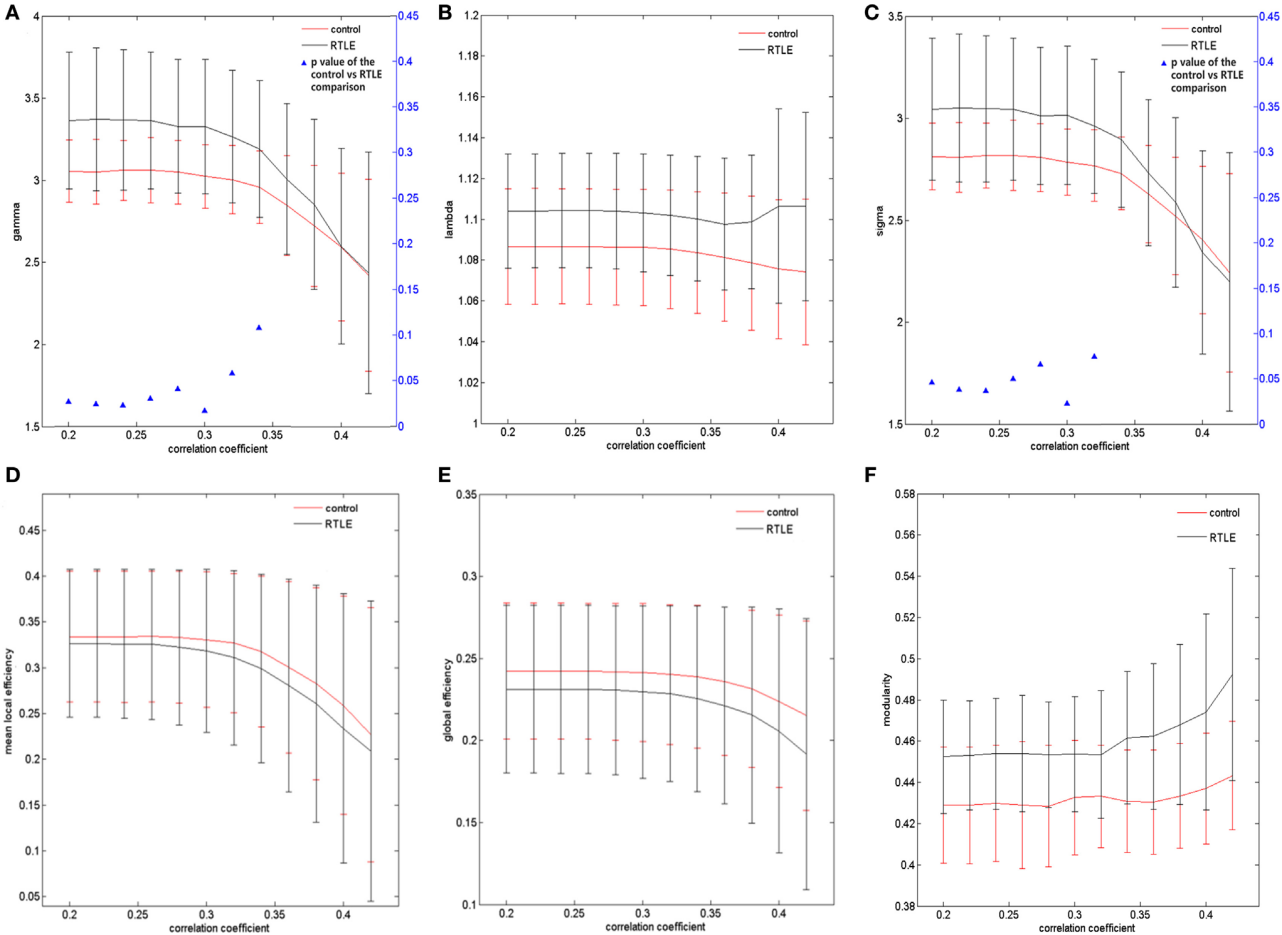
**Structural connectivity network at different FA threshold for right temporal lobe epilepsy (rTLE) patients (the black line) and controls (the red line) and their statistical comparison results (ANCOVA)**. **(A)** Gamma, **(B)** lambda, **(C)** sigma, **(D)** mean local efficiency, **(E)** global efficiency, **(F)** modularity. Error bars indicate standard error within each group at each threshold value. The blue triangles display the (ANCOVA) *p*-values.

The graph measurements of the DTI network were computed over a series of thresholds on FA values (FA = 0.2~0.42) with a step of 0.02. Both rTLE patients and healthy controls showed a small-world organization (σ > 1, with λ close to 1 and γ higher than 1) (Figures 1A,B). Compared with healthy controls, rTLE patients had a higher clustering coefficient (γ) (Figure 2A, FA ≤ 0.28, *p* < 0.05) and the same characteristic path length (λ) (Figure 2B), which led to significantly elevated small-worldness (σ) (Figure 2C, FA ≤ 0.26 and FA = 0.30). Since σ reflects an optimal balance between fragmentation and coalescence, our results indicate that there is a disturbance in the normal balance of network function. Therefore, network construction was more inclined to regular network characteristics. In addition, rTLE showed unchanged mean local efficiency (E_loc_) (Figure 2D) and global efficiency (E_glob_) (Figure 2E).

**Figure 3:**
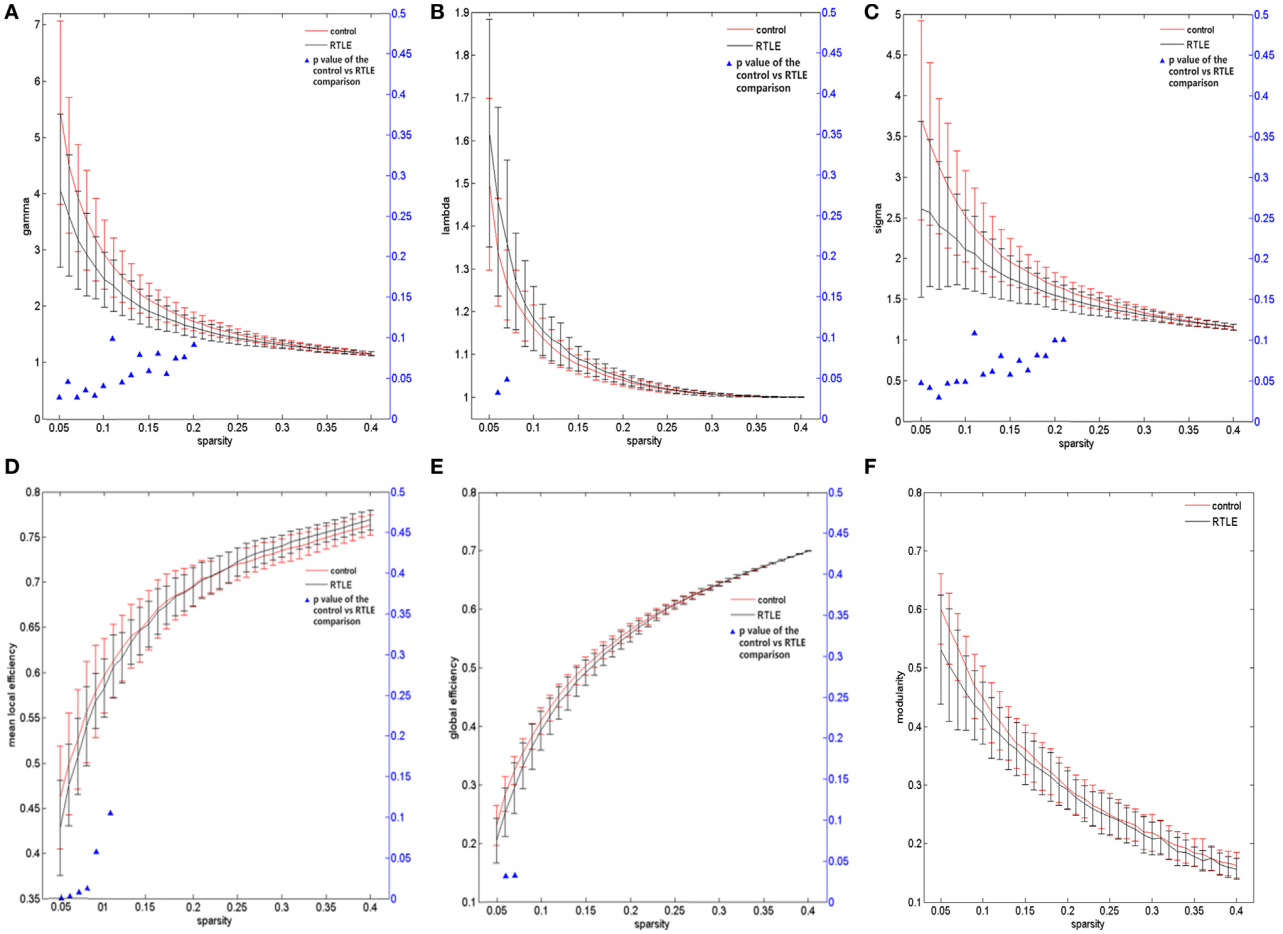
**Functional connectivity network at different sparsity for right temporal lobe epilepsy (rTLE) patients (the black line) and controls (the red line) and their statistical comparison results (ANCOVA)**. **(A)** Gamma, **(B)** lambda, **(C)** sigma, **(D)** mean local efficiency, **(E)** global efficiency, **(F)** modularity. Error bars indicate standard error within each group at each threshold value. The blue triangles display the (ANCOVA) *p*-values.

For the rsfMRI datasets, the correlation matrix was thresholded into different sparsities to create the adjacency matrix. Since there is currently no definitive method of selecting a sole threshold, we calculated graph measures of the rsfMRI network over a series of thresholds in a wide range of network sparsity (5~40%) with a step of 1% (Figures 1C,D). Compared with healthy controls, rTLE patients had s lower γ (Figure 3A, sparsity <11%) and higher λ (Figure 3B, 5% < sparsity < 8%), which led to s significantly reduced σ (Figure 3C, sparsity <11%). As a result of these analyses, the FC network architecture tended to a more random organization. After controlling for the FC strength difference using the sparsity threshold, rTLE showed lower Eloc (Figure 3D, sparsity <9%), and lower Eglob (Figure 3E, 5% < sparsity < 8%).

We also analyzed the modularity of the structural and functional networks of the two groups, and there was no significant difference (Figures 2F and 3F).

### ANT Results of Patients and Controls

Alertness did not differ significantly between the rTLE patients and controls. However, the RTs of no cue and double cue of rTLE patients were significantly longer than those of controls. Table 2 provides more details.

**Table 2:** **Neuropsychological attention network test test performance of right temporal lobe epilepsy (rTLE) patients and healthy controls**.

Characteristics	rTLE (*n* = 18)	Control (*n* = 19)	*t* Value	*p*-Value
RT_no-cue_ (ms)	655.55 ± 98.49	594.70 ± 82.60*	2.041	0.049
RT_double-cue_ (ms)	614.99 ± 95.16	549.44 ± 73.63*	2.351	0.024
Alertness (ms)	40.56 ± 23.91	45.25 ± 18.63	0.668	0.508

*Values: the Mean ± SD*.

*p < 0.05.

### Association of the Global Topological Parameters and Alertness

We analyzed the correlations between alertness and altered global topological parameters of the structural and functional networks in rTLE patients at each threshold value. For their structural networks, we found that σ and γ were significantly negatively correlated with alertness in the FA value range (0.34 < FA < 0.42) (Table 3). Similarly, after controlling for disease duration as a confounding variable, partial correlation analysis showed the same trend in the FA value range (0.36 < FA < 0.42) (Table 3). No correlation was found in Pearson correlation or partial correlation analyses of alertness with any other parameters.

**Table 3:** **Pearson correlation analysis and partial correlation analysis (disease duration-corrected) between the topological characteristics of the structural network and alertness in rTLE patients**.

Properties	FA threshold	Values	Pearson correlation analysis		Partial correlation analysis	
			*r*-Value	*p*-Value	*r*-Value	*p*-Value
Sigma	0.34	2.90 ± 0.33	−0.47	0.049	–	–
	0.36	2.73 ± 0.36	−0.539	0.021	−0.53	0.028
	0.38	2.59 ± 0.42	−0.612	0.007	−0.606	0.01
	0.4	2.34 ± 0.50	−0.532	0.023	−0.549	0.022
	0.42	2.20 ± 0.63	−0.604	0.008	−0.606	0.01
Gamma	0.34	3.19 ± 0.42	−0.486	0.041	−0.488	0.047
	0.36	3.00 ± 0.46	−0.524	0.025	−0.517	0.034
	0.38	2.85 ± 0.52	−0.583	0.011	−0.577	0.015
	0.4	2.59 ± 0.60	−0.518	0.028	−0.531	0.028
	0.42	2.43 ± 0.74	−0.589	0.01	−0.589	0.013
Lambda	All threshold	1.42 ± 0.04	–	–	–	–
E_loc_	All threshold	0.23 ± 0.05	–	–	–	–
E_glob_	All threshold	0.33 ± 0.08	–	–	–	–

*Values: the mean ± SD*.

*FA, fiber fractional anisotropy*.

*Correlation analysis showed a negative correlation between the sigma (σ) and gamma (γ) of the functional network and alertness in right temporal lobe epilepsy (rTLE) patients over the FA threshold range described in the table (*p* < 0.05, Pearson correlation analysis and Partial correlation analysis)*.

For the functional networks, σ and γ were also found to be significantly negatively correlated with alertness over a range of sparsity thresholds (for σ, 0.28 < sparsity < 0.40; for γ, 0.26 < sparsity < 0.40; *p* < 0.05), as analyzed by the Pearson correlation and partial correlation analyses. E_loc_ had no correlation with the ANT scores by Pearson correlation analysis, but was negatively correlated with alertness at several different sparsity thresholds (sparsity = 0.17, 0.24, and 0.25; *p* < 0.05) when calculated by partial correlation analysis and controlling for the influence of the disease duration. However, no correlation was found between λ, E_glob_ and alertness when the above two methods were used (Table 4). In the control group, there was no association between ANT scores and any network parameter.

**Table 4:** **Pearson correlation analysis and partial correlation analysis (disease duration-corrected) between the topological characteristics of the functional network and alertness in rTLE patients**.

Properties	Sparsity	Values	Pearson correlation analysis		Partial correlation analysis	
			*r*-Value	*p*-Value	*r*-Value	*p*-Value
Sigma	0.26	1.38 ± 0.10	–	–	−0.495	0.044
	0.27	1.36 ± 0.09	–	–	−0.502	0.04
	0.28	1.34 ± 0.08	0.496	0.036	−0.531	0.028
	0.29	1.32 ± 0.08	−0.496	0.036	−0.52	0.032
	0.3	1.30 ± 0.07	−0.488	0.04	−0.507	0.038
	0.31	1.29 ± 0.06	−0.533	0.023	−0.549	0.022
	0.32	1.27 ± 0.06	−0.051	0.034	−0.536	0.027
	0.33	1.25 ± 0.05	−0.544	0.02	−0.579	0.015
	0.34	1.24 ± 0.05	−0.572	0.013	−0.613	0.009
	0.35	1.22 ± 0.05	−0.529	0.024	−0.58	0.015
	0.36	1.20 ± 0.04	−0.526	0.025	−0.577	0.015
	0.37	1.19 ± 0.04	−0.514	0.029	−0.574	0.016
	0.38	1.18 ± 0.04	−0.487	0.04	−0.547	0.023
	0.39	1.17 ± 0.04	−0.486	0.041	−0.55	0.022
	0.4	1.16 ± 0.03	−0.483	0.043	−0.554	0.021
Gamma	0.26	1.40 ± 0.10	−0.478	0.045	−0.52	0.032
	0.27	1.38 ± 0.09	−0.482	0.043	−0.525	0.031
	0.28	1.36 ± 0.08	−0.511	0.03	−0.552	0.022
	0.29	1.33 ± 0.08	−0.515	0.029	−0.538	0.026
	0.3	1.31 ± 0.07	−0.505	0.032	−0.528	0.03
	0.31	1.29 ± 0.06	−0.547	0.019	−0.565	0.018
	0.32	1.28 ± 0.06	−0.512	0.03	−0.551	0.022
	0.33	1.25 ± 0.05	−0.55	0.018	−0.589	0.013
	0.34	1.24 ± 0.05	−0.576	0.012	−0.622	0.008
	0.35	1.22 ± 0.05	−0.535	0.022	−0.59	0.013
	0.36	1.21 ± 0.04	−0.529	0.024	−0.585	0.014
	0.37	1.19 ± 0.04	−0.515	0.029	−0.578	0.015
	0.38	1.18 ± 0.04	−0.489	0.039	−0.551	0.022
	0.39	1.17 ± 0.04	−0.488	0.04	−0.551	0.021
	0.4	1.16 ± 0.03	−0.483	0.042	−0.557	0.02
Lambda	All sparsity	1.15 ± 0.06	–	–	–	–
E_loc_	0.17	0.67 ± 0.02	–	–	−0.534	0.027
	0.24	0.72 ± 0.02	–	–	−0.512	0.035
	0.25	0.72 ± 0.02	–	–	−0.488	0.047
E_glob_	All sparsity	0.44 ± 0.03	–	–	–	

*Values: the mean ± SD*.

*Correlation analysis showed negative correlation between the sigma (σ) and gamma (γ) of the functional network and alertness in right temporal lobe epilepsy (rTLE) patients over the sparsity range describing in the table (*p* < 0.05, Pearson correlation analysis and Partial correlation analysis). Moreover, partial correlation analysis showed a negative correlation between local efficiency (E_loc_) and alertness when sparsity corresponded to 0.17, 0.24, and 0.25*.

## Discussion

In the present study, we compared the global alterations of the network properties in parents with rTLE to those of healthy controls by using structural and FC and graph theoretical techniques. Both structural networks and functional networks of all subjects showed a prominent small-world property (with σ > 1, a higher clustering coefficient but a lower characteristic path length). The main results indicated that there were global alterations of network properties in rTLE, including structural networks that trended toward regular alterations and functional networks that trended toward random alterations. The changes of the topological properties of the function network were much greater than those of the structure network in rTLE patients. In addition, association analyses showed that alertness had a negative correlation with the clustering coefficient and small-worldness properties in rTLE patients.

### Network Properties

At this time, DTI is the only non-invasive technique that provides tissue microstructural information *in vivo*. The DTI-based structural linkage provides a relatively intuitive method of describing the true structural network between brain regions (36). Many neuroimaging studies have shown that TLE is associated with structural abnormalities in specific brain areas. Subsequent findings of white matter damage in or outside the temporal lobe were reported in TLE, including white matter damage in the external capsule, corpus callosum, cingulate gyrus, and hook beam, as well as the amount of the next pillow beam (37). These findings have ignited interest in white matter network structure studies in TLE that may identify experimental targets of pathophysiology mechanisms. In this study, we found that rTLE patients, compared to healthy controls, exhibited an altered topological organization of the white matter structure network and, based on DTI tractography, these alterations included highly increased clustering coefficients and small-worldness.

The clustering coefficient represents the small range of connections between adjacent brain areas, which is used to describe the capability to effectively interchange information and information reprocessing in a closed feedback loop and expresses the constitute forms of cliquishness within network (38, 39). A higher clustering coefficient often indicates modularized information processing and an enhancement of the local specialization and separation of network functionally (38). At present, the increased clustering coefficient and small-worldness could be interpreted as alterations of an efficiently organized network. Our results suggest that microstructure damage might occur in white matter structures because of recurrent, uncontrolled seizures. For instance, subtle alterations in white matter tract volumes were determined to be due to transneuronal degeneration in a diffuse underlying pathology, such as microdysgenesis (2). Aberrant local nerve fibers may be reconstructed as a compensatory mechanism in response to a decrease in long-range connections, such that the relatively high reentrant connectivity within the local clusters (“cliquishness”) increased. In this way, the efficiency of the entire brain network is preserved.

Unlike structural networks, we found significantly decreased clustering coefficients and enhanced shortest path lengths, as well as lower local and global efficiencies in brain functional networks. Our findings are consistent with those of Vlooswijk et al.’s (40). In studies of functional networks in TLE, the clustering coefficients have been widely reported to be either increased (3, 41, 42) or decreased (40, 43, 44). The reasons for these inconsistent findings are unclear, although it is important to account for the sample size, age, epilepsy phenotype, measurement of the connection form, and AED use. A lower clustering coefficient tends to indicate a weakening interconnection between local brain regions, suggesting a possible reduction in the separation function of brain processing information associated with a particular pathological condition. Some authors have observed that the clustering coefficient also changes during the progression of disease. Evidence from a graph theory study of neuron sclerosis in the dentate gyrus showed that the clustering coefficients increased during the sclerosis process and decreased at the final stage (45). In combination with the enhanced shortest path lengths, which indicated that the global integration of the brain was decreased, that is, information transmissions or interactions between remote brain regions were less effective and slow. Therefore, the global information progressing efficiency of the brain functional network of rTLE patients was significantly diminished. Epileptic seizures are due to the synchronous excitability of abnormal neurons in the brain, such as a high synchronization between thalamus and remote cortical regions (46), and increased EEG connection between relevant brain area to the epileptic foci (7), etc. Changes of the above properties may be related to enhanced neural synchronization phenomena. Consequently, high synchronization within a group of neurons in the brain often leads to a decline in global brain function.

The inconsistency of the alteration of the network topology characteristics between structural and functional networks may result from the following reasons. In general, functional networks are considered to be more resilient, while structured networks are considered to be relatively stable (47). We presumed that functional networks are probably more sensitive than structural networks and undergo dynamic and architecture changes at earlier stages of the disease. Yet, structural networks are affected during later stages of the disease. White matter organization is rebuilt to ensure proper brain function, and this reorganization is a compensatory mechanism of brain plasticity. It is also possible that reconstructions of the structure network are under restrictions (48). The compensatory ability of functional networks is limited by the white matter axon plexus structure, and because of these limits, the network efficiency decreases.

### Correlation between Alertness and Topology in Structural and Functional Networks

Mental and behavioral disorders caused by TLE have recently become a prominent research topic. Alertness is one of the three sub-networks of attention, but it is often overlooked and rarely reported. Alertness consists of two components: intrinsic alertness and phasic alertness. Intrinsic alertness is associated with the body’s internal alert and arousal states. It can respond to the target stimulus without external stimulate signals. Phasic alertness represents a short-term improvement in response to external stimuli. Both of these are measured by the RTs in the ANT test. The alertness that we obtained is the difference between the RTs of two parts (49). Although no significant differences in alertness were found between rTLE patients and the controls, the RTs of no cue and double cue of patients were longer than those of controls. Furthermore, one of our other researchers discovered longer RT_no-cue_, RT_double-cue_ and lower alertness as well (50). Together, these data demonstrate a potential alertness impairment in TLE patients.

According to the association analysis, there was a negative correlation between alertness and the clustering coefficient and the small-worldness property, both in white matter structural networks and in functional networks. For structural networks, these data suggest that the potential decline in alertness might be related to the increase of these two properties. It is likely that repeated seizures injured the microstructure of white matter fibers. Consequently, white matter structure remodeling occurred as a consequence of brain plasticity. Thus, structural networks enhance localized processing to maintain effective interactions in information flow. Similarly, Bonilha et al.’s study indicated that neuronal loss caused by seizures theoretically leads to reorganization of the limbic system (3). Therefore, limbic system reorganization could lead to neuropsychological abnormalities in TLE. As to the correlation in functional networks described above, we presumed that alertness is associated with the degree of global integration of brain functional networks. Further, it means that alertness may be a function for which multiple brain regions process information in parallel and concurrently, rather than as a single group of neurons or a single neuronal circuit. As part of the compensation mechanism of functional networks, reduced local specificity and enhanced functional collaborations maintain the patient’s alertness level. Therefore, differences in clinical behavioral deficits would not be obvious.

In addition, taking into account the possible effects of the disease duration of epilepsy, we used partial correlation analysis to correct for the impact of the length of disease. Most of the results were consistent. However, a negative correlation between alertness and the local efficiency of functional networks was observed, although the negative correlation only occurred over a narrow range of sparsity thresholds. Combined with the non-correlation between alertness and the λ and E_glob_ of functional networks, the possible explanation would be that, although the network efficiency decreased, both the decline in functional separation and increase in global integration maintained alertness. Therefore, the rTLE patients in our study had no temporarily clinically detected significant alertness deterioration. The disease duration has little involvement in the relationship between alertness and network topology.

In this study, there are several limitations that are worth noting. Graphical analysis of complex neural networks at the macro-scale level is a fast-growing area of research, but there is still controversy regarding the optimal analytic strategies. Node definition and selection are very important issues in building structural or functional networks (51). In addition, the cross-sectional design of this study limited our ability to expose the mechanism of the causal relationship between the network analysis results and clinical behavioral testing. By choosing nodes according to different brain anatomical templates in further studies, we will be able to find more complex information processing mechanisms of the brain.

## Author Contributions

WJ was responsible for conceiving and designing the study as well as writing the manuscript. JL performed data analysis and statistical processing. XC was in charge of data acquisition and management. WY provided and integrated neuroimaging data. JZ was the head of funding and supervised the paper. All of the authors approved the final version of the manuscript.

## Conflict of Interest Statement

The authors declare that the research was conducted in the absence of any commercial or financial relationships that could be construed as a potential conflict of interest.

## Footnotes

^1^http://www.nitrc.org/projects/Panda

^2^http://www.fil.ion.ucl.ac.uk/spm

^3^www.brain-connectivity-toolbox.net/

^4^http://vlado.fmf.uni-lj.si/pub/networks/pajek/
